# Microarray studies on the effect of silencing *tynA* in *Escherichia coli* K-12

**DOI:** 10.1016/j.gdata.2015.12.018

**Published:** 2015-12-28

**Authors:** Heli Elovaara

**Affiliations:** Medicity Research Laboratory, University of Turku, Turku, Finland; Department of Medical Microbiology and Immunology, University of Turku, Turku, Finland

**Keywords:** *Escherichia coli*, *tynA*, Primary amine oxidase, Microarray, ECAO

## Abstract

To study the biological role of the *tynA* gene product of *Escherichia coli*, a primary amine oxidase (ECAO, E.C. 1.4.3.21), the *tynA* gene was genetically silenced by conjugation with a kanamycin resistance cassette. We used a microarray method to compare the mRNA expression in the modified strain (Δ*tynA*) to that in the wild type (*wt*) strain at the time of induction of ECAO expression (0 h) as well as 1 h and 4 h after the induction. These data in brief describe the different experimental conditions, sample preparation, data collection and analysis of the conducted microarray experiment. The differential expression of genes in the studied strains 1 h after the induction of ECAO expression is described. The microarray data have been deposited in NCBI's Gene Expression Omnibus and are accessible through GEO Series accession number GSE65385.

**Table:** 

Specifications	
Organism/cell line/tissue	*Escherichia coli* K-12
Sex	*N*/*A*
Sequencer or array type	*Affymetrix* GeneChip *E. coli* Genome 2.0 Array
Data format	*Raw* or *analyzed*
Experimental factors	*tynA* deletion mutant *Escherichia coli* K-12 and *wt* control
Experimental features	Identify the gene expression changes in *Escherichia coli* bacteria lacking a *tynA* gene.
Consent	*N*/*A*
Sample source location	Finland, a sepsis isolate

## 1 Direct link to deposited data

(http://www.ncbi.nlm.nih.gov/geo/query/acc.cgi?acc=GSE65385). Accession number GSE65385.

## 2 Experimental design, materials and methods

*Escherichia coli* (*E. coli*) K-12 harbors a *tynA* gene, which encodes for a periplasmic primary amine oxidase (ECAO, E.C. 1.4.3.21). We constructed a genetically modified *E. coli* K-12 strain unable to express ECAO (Δ*tynA*, [1]). Both wild type (wt) and Δ*tynA* strains were cultured in conditions, which induce the expression of ECAO, to be able to compare the expression profiles of Δ*tynA* and *wt* bacteria. We analyzed the samples at the start of the induction as well as 1 h and 4 h after the induction. We have published a research article describing the construction of the Δ*tynA* strain together with the full interpretation of the effect of the deletion of *tynA* on the expression of genes at the 4 h time point [1]. Here the methods used are described in more detail as well as the list of differentially expressed genes at 1 h after the induction of ECAO is presented.

### 2.1 Constructing the Δ*tynA* strain

Detailed information about the generation of the modified Δ*tynA* strain is published [1]. In brief, we introduced a kanamycin cassette into the *tynA* gene by homologous recombination utilizing a bacterial conjugation to silence the gene.

### 2.2 Bacterial culture conditions

For the microarray sample preparation, we inoculated *wt* and Δ*tynA* bacteria in 5 ml of modified M9-lactose medium (0.2 M Na_2_HPO_4_, 0.2 M KH_2_PO_4_, 90 mM NaCl, 0.2 M NH_4_Cl, 1 mM MgSO_4_, 0.2% lactose, 0.1 mM CaCl_2_, and 1 mM Thiamine–HCl) at 30 °C, 250 rpm, and 16 h. Then 0.5 ml of bacterial culture was withdrawn for the RNA extraction to be used as the control sample for the induction (0 h, see below). The rest of the culture was renewed in a fresh, pre-warmed modified M9-lactose medium supplemented with 0.5 mM CuSO_4_ and 5 mM phenylethylamine (PEA) 1:20 to induce the expression of the *tynA* gene. Bacterial cultures were then further cultivated at 30 °C and 250 rpm. The bacterial samples (0.5 ml) were withdrawn for the RNA extraction at 1 and 4 h (A_600_ = 0.3) after the induction. After the withdrawal of the samples, 1 ml of RNAprotect reagent (Qiagen) was added to every sample. The bacterial culture samples were stored at − 20 °C before the extraction of RNA.

### 2.3 RNA extraction

The total RNA was extracted by RNeasy Mini-Kit (Qiagen). DNAs of the samples were excised by RNase-Free Dnase Set (Qiagen). NanoDrop ND-1000 (Thermo Scientific) was used for the determination of the RNA concentration. The quality of the RNA was assayed with the BioRad's Experion electrophoresis station and Bioanalyzer. The good quality RNA has only major peaks of 18S and 28S (ratio 1:2; GeneChip Expression Analysis Data Analysis Fundamentals, Affymetrix), and by observing A_260_/A_280_ = 1.8–2.1. Two of the samples had the ratio slightly above the desired 2.1 (2.2 and 2.3, respectively). However, we used the pool of seven samples for each experimental condition, and therefore also these two samples were used because the ratios of other samples in the pool were within the desired range.

### 2.4 cDNA preparation and array hybridization

The sample cDNA preparation, biotin labeling, sample hybridization, and microarray studies were all conducted at the Finnish DNA Microarray Centre at Turku Centre for Biotechnology, Turku, Finland. For the cDNA preparation we pooled identical amounts of seven different samples of each condition (0 h, 1 h and 4 h) to yield the required 10 μg of RNA (the analysis was conducted at 2008). Samples were processed according to the Prokaryotic Target Preparation protocol from GeneChip® Expression Analysis Technical Manual (with specific protocols for using the GeneChip® Hybridization, Wash and Stain Kit; [2]). No replicates were made.

cDNA was labeled according to Affymetrix based end-terminus labeling with biotin [2]. A total of 3 μg of labeled cDNA was used for the fragmentation reaction for each experiment. The quality of the fragmentation and labeling was checked with a gel-shift assay [2].

Biotinylated cDNA fragments, 2.4 μg for each experimental condition, were hybridized to GeneChip *E. coli* Genome 2.0 Array at 45 °C for 16 h according to the GeneChip Expression Analysis Technical Manual [2]. The success of hybridization was controlled by the increasing signals for the spiked *bioB*, *bioC* and *bioD* from *E. coli* and *cre* of *P1 bacteriophage* anti-sense biotinylated cRNA-probes [2].

Hybridized arrays were stained with streptavidin and washed according to the GeneChip Expression Analysis Technical Manual [2]. The staining protocol was controlled by the observation that the spiked RNA probe set (*lys*, *phe*, *thr* and *dap* genes from *Bacillus subtilis*) was present in the increasing signal values [2]. The staining and washing steps were conducted by the GeneChip Fluidics Station 450, which was controlled by the Affymetrix GeneChip Command Console (AGCC) software version 1.0 [2]. After the washes the arrays were scanned with GeneChip Scanner 3000 with an AutoLoader. The quality was controlled by the Affymetrix Expression Console. AGCC generated CEL files automatically. The CEL files are available under the accession number GSE65385 in the GEO database.

### 2.5 Microarray data analysis

Data analysis was performed using the R language and environment for statistical computing and bioconductor [3], [4]. We normalized the data using the gcRMA method [5], after which the intensity distribution of the samples was nearly identical (data not shown). The statistical comparison between the samples was carried out using the Limma package [6]. The differentially expressed genes were filtered requiring absolute fold-change of at least 2.5. Additionally, a mean absolute expression value of at least 3.0 and a minimum difference of 3.0 were required between the compared samples.

## 3 Results

This report describes the detailed sample preparation conditions together with the details of the microarray experiment and analysis. In our experimental setup we compared the expression of genes of *wt E. coli* K-12 and its genetically modified Δ*tynA* strain at time points 0 h, 1 h and 4 h. The analysis of differentially expressed genes 4 h after induction has been published in our primary paper [1]. Here we briefly describe the differences between two strains 1 h after the induction of ECAO expression, which was not described in our primary publication.

At the start of the induction of the *tynA* gene (0 h), we detected no difference between *tynA* gene expression between the wild type and Δ*tynA* strains (normalized expression level of *tynA* was 2.48 and 2.68 for the *wt* and *tynA* strains, respectively). Because we were only interested in the effect of the lack of *tynA* expression, we did not analyze the differentially expressed genes at this time point. However, the existing data are freely available (see link to the deposited data).

As expected, the transfer of bacteria to PEA containing medium led to the increasing expression of *tynA* in the *wt* strain as a function of time (normalized expression levels were 37.73 and 422.78 from the time point 1 h to 4 h, respectively), meanwhile the normalized expression in the Δ*tynA* remained at background level (2.04 and 1.98, respectively). When the gene expression of the Δ*tynA* strain was compared to the *wt* strain at the 1 h time point, we detected the down-regulation of only a few genes above the threshold of − 2.5 fold change (FC, Table 1). In addition to our target gene, *tynA*, the gene encoding for the next enzyme in the PEA pathway, *feaB*, was also down-regulated (Table 1). However, in comparison to the situation at the 4 h time point, where we were able to see the down-regulation of the whole phenylethylamine utilization pathway, we detected no down-regulation of genes in the *paaABCDEFGHIJKXY* operon [1]. In contrast, we observed a slight down-regulation of *fhuF*, ferric ion reductase, *wcaJ*, a putative UDP-glucose lipid carrier transferase and one hypothetical protein, which were not down-regulated 4 h after the induction (Table 1). The differences in the differentially expressed genes between 1 h and 4 h after the induction was not surprising, as the activity of ECAO reached a plateau 2 h after the induction, and was at the 1 h time point less than 25% of the activity at 4 h after the induction [1].

In the list of up-regulated genes, we observed only five genes, whose expression was up-regulated in the Δ*tynA* strain 1 h after the induction when compared to the *wt* strain (Table 2). The FC values remained low, under 3.5, and none of the up-regulated genes at this time point was up-regulated at the 4 h time point.

## Figures and Tables

**Table 1:** Differentially down-regulated (FC < − 2.5) genes in Δ*tynA* vs. *wt E. coli* 1 h after *tynA* induction*.* According to the David functional annotation analysis the gene list of down-regulated genes at time point 1 h was specifically associated (p-value < 0.05) with pathways of phenylalanine metabolism and biosynthesis of siderophore group nonribosomal peptide.

Gene symbol	Gene	FC
*tynA*	Tyramine oxide, copper containing	− 19.49
*feaB*	Phenylacetaldehyde dehydrogenase	− 4.08
*fhuF*	Ferric ion reductase involved in ferric hydroximate transport	− 2.97
*c0651*	Hypothetical protein ybcY precursor	− 2.68
*wcaJ*	Putative UDP-glucose lipid carrier transferase	− 2.62

**Table 2:** Differentially up-regulated (FC > 2.50) genes in Δ*tynA* vs. *wt E. coli* 1 h after the induction of ECAO expression.

Gene symbol	Gene	FC
*ydiE*	Hypothetical protein	3.13
*tnaC*	Tryptonase leader peptide	3.07
*metN*	dl-Methionine transporter ATP-binding subunit	2.88
*c2375*	Hypothetical protein	2.83
*yddM*	Predicted DNA-binding transcriptional regulator	2.57
